# Morphology of ovaries in laron dwarf mice, with low circulating plasma levels of insulin-like growth factor-1 (IGF-1), and in bovine GH-transgenic mice, with high circulating plasma levels of IGF-1

**DOI:** 10.1186/1757-2215-5-18

**Published:** 2012-07-02

**Authors:** Sylwia Słuczanowska-Głąbowska, Maria Laszczyńska, Katarzyna Piotrowska, Wojciech Głąbowski, John J Kopchick, Andrzej Bartke, Magda Kucia, Mariusz Z Ratajczak

**Affiliations:** 1 Department of Physiology, Pomeranian Medical University, Powstańców Wielkopolskich 72, 70-111, Szczecin, Poland; 2 Department of Histology and Developmental Biology, Pomeranian Medical University, Żołnierska 48, 71-210, Szczecin, Poland; 3 Department of Histology and Embryology, Pomeranian Medical University, Powstańców Wielkopolskich 72, 70-111, Szczecin, Poland; 4 Edison Biotechnology Institute and Department of Biomedical Sciences, College of Osteopathic Medicine, Ohio University, Athens, OH, USA; 5 Geriatrics Research, Departments of Internal Medicine and Physiology, Southern Illinois University School of Medicine, Springfield, IL, USA; 6 Stem Cell Biology Program, James Graham Brown Cancer Center, University of Louisville, Louisville, Ky, USA

**Keywords:** Murine ovary, Laron dwarf mouse, Bovine growth hormone transgenic mouse, Growth hormone, Insulin-like growth factor-1, Aging

## Abstract

**Background:**

It is well known that somatotrophic/insulin signaling affects lifespan in experimental animals, and one of the signs of aging is progressive gonadal dysfunction.

**Methods:**

To study the effects of insulin-like growth factor-1 (IGF-1) plasma level on ovaries, we analyzed ovaries isolated from 2-year-old growth hormone receptor knockout (GHR-KO) Laron dwarf mice, with low circulating plasma levels of IGF-1, and 6-month-old bovine growth hormone transgenic (bGHTg) mice, with high circulating plasma levels of IGF-1. The ages of the Laron dwarf mutants employed in our studies were selected based on their overall survival (up to _~_ 4 years for Laron dwarf mice and _~_ 1 year for bGHTg mice).

**Results:**

Morphological analysis of the ovaries of mice that reached _~_50% of their maximal life span revealed a lower biological age for the ovaries isolated from 2-year-old Laron dwarf mice than their normal-lifespan wild type littermates. By contrast, the ovarian morphology of increased in size 6 month old bGHTg mice was generally normal.

**Conclusion:**

Ovaries isolated from 2-year-old Laron dwarf mice exhibit a lower biological age compared with ovaries from normal WT littermates at the same age. At the same time, no morphological features of accelerated aging were found in 0.5-year-old bGHTg mice compared with ovaries from normal the same age-matched WT littermates.

## Introduction

Senescence is a physiological process related to changes in many tissues and organs, including dysfunction of the endocrine system, and among the first changes observed are a decrease in growth hormone (GH) and sex hormone levels in plasma. It is well documented that serum levels of GH decline with age in both mouse and human, and several murine models have been identified as potential models for the role of GH in aging [1-3].

GH circulating in plasma stimulates liver secretion of somatomedin-C, also known as insulin-like growth factor -I (IGF-1), which affects the function of several organs, including the gonads. Studies in mice deficient in GH, its receptor GHR, IGF-I, or IGF-I receptor (IGF-1R) revealed that GH/IGF-I signaling is required for the normal rate of sexual development and maturation [4-7]. GH and IGF-I act on all levels of the hypothalamic–pituitary–gonadal axis (HPG) and regulate the function of the reproductive system and mammary glands. Specifically, GH/IGF-I signaling affects (i) release of gonadotropin-releasing hormone (GnRH) and gonadotropins, (ii) expression of receptors for gonadotropins in ovarian granulosa cells and in Leydig cells in testes, and (iii) development of mammary glands. GH can also temporarily mimic the function of gonadotropins, as previously reported [4,8,9].

The GH/IGF-I axis is crucial for development and maturation of ovarian follicles. GH also controls early phases of follicle development and stimulates formation of secondary follicles and the development of granulosa and theca cells. GH initiates the growth of primordial follicles and supports the development of primary and secondary follicles. Silva et al. [9] suggest that GH is the survival factor for primary follicles and regulates differentiation of granulosa cells. GH also augments IGF-1 secretion by granulosa and theca cells, steroidogenesis in granulosa cells, and development and maturation of oocytes [9]. It has also been shown that GH is responsible for maintaining the sensitivity of granulosa cells to gonadotropins. On the other hand, gonad-derived sex steroids enhance the release of GH, and synergy between sex steroids and GH promotes development and maturation of the follicles [4,6,9-11].

It is well known that IGF-I is crucial for fertility and IGF-I knockout mice have been reported to be sterile [12]. There are two sources of IGF-I that affect the function of ovaries i) IGF-1 released from liver into the circulating blood plasma in response to stimulation by GH and ii) IGF-1 locally produced in tissues, including the gonads. The IGF-I receptor (IGF-IR) has been reported to be present on granulosa cells in most mammals and is also expressed on rat oocytes [13]. In ovaries, the IGF-I/IGF-1R axis i) activates development of preantral follicles, ii) maintains the larger pool of small antral follicles, iii) stimulates the development of follicles, iv) selects dominant follicles, and v) stimulates steroidogenesis in theca cells and secretion of progesterone by large antral follicles [9,14]. Danilovich et al. [15] demonstrated that bovine growth hormone (bGH)-expressing transgenic mice display a decrease in athreticpreantral follicles, which suggests that GH or IGF-I prevents apoptosis of granulosa cells.

The bioavailability of IGFs is regulated by a family of intrafollicular-expressed IGF binding proteins (IGFBPs) [13,14,16]. Wandji et al. [16] analyzed the expression of IGFBPs in different stages of development and atresia of ovarian follicles. The high levels of these proteins has been observed during early development of follicles, and the decrease in IGFBP level leads to an increase in IGF-1 bioavailability, which stimulates proliferation of granulosa cells and steroidogenesis [13,14].

The importance of GH/IGF-I signaling in reproduction has been investigated in different experimental animals. As mentioned above, while IGF-I knockout (IGF-1-KO) mice are sterile, GH receptor-knockout (GHR-KO) Laron dwarf mice are fertile [4,5,12]. Laron syndrome is an inherited, recessive disorder related to GH resistance and is characterized by high plasma GH levels and severely reduced levels of plasma-circulating IGF-I. An animal model of human Laron syndrome [5] has been created by targeted disruption of the growth hormone receptor binding protein (GHR/BP) gene, which significantly impairs GH-mediated release of IGF-I from liver. Female Laron dwarf mice were reported to have delayed sexual maturation, which is evident by the advanced maternal age at first conception. While the luteinizing hormone (LH) response to stimulation by gonadotropin-releasing hormone (GnRH) and secretion of follicle stimulating hormone (FSH) secretion are reduced in these mice, the prolactin (PRL) level is increased. At the morphological level, a reduction in the numbers of preovulatory follicles and corpora lutea has been found [5,10].

The influence of GH on the female reproductive system has also been studied in transgenic mice that overexpress bovine (b) bGH (bGHTg mice), which represent an opposite endocrine endocrine phenotype than the long-living murine mutants with reduced activity of the GH/IGF-1 axis (e.g., Laron dwarf mice). Increases in GH/IGF-1 signaling lead to increases in body mass, organomegaly, and reduction in adipose tissue. The lifespan of bGH mice is reduced[17-20], puberty accelerated, the ovulation rate increased, and yet fertility is reduced proportional to the increase in plasma GH levels [21,22].

Although, there are several reports about the physiological effects of GH/IGF-1 signaling on the murine reproductive system, including in Laron dwarf and bGHTg mice, studies on ovarian morphology have not been performed. Thus, the aim of this study was to compare ovarian morphology between 2-year-old Laron dwarf mice and 2-year-old WT littermates, as well as between 0.5-year-old bGHTg animals and similarly aged WT littermates. The age of Laron dwarf and bGHTg animals employed in this study corresponded to approximately the midpoint of their life span.

## Material and methods

### Animals

Mice were bred at the animal facility at Southern Illinois University Medical School and given free access to nutritionally balanced diet and tap water. The experiments were performed on female adult mice divided into four groups. Accordingly, we compared 2 years old *Laron dwarf (GHR*^*–/–*^*)* mice (n = 11) to2 years old wild type (WT) mice (n = 11) and 6 month old bovine GH transgenic mice (bGHTg) (n-5) to wild type (WT) mice (n = 5) at the same age.

This study was performed in accordance with the guidelines of the Animal Care and Use Committee of the University of the Southern Illinois University Laboratory Animal Care Committee and University of Louisville School of Medicine and with the Guide for the Care and Use of Laboratory Animals (Department of Health and Human Services, publication no. NIH 86-23).

#### Laron dwarf (GHR^–/–^) mice

Control and GHR^–/–^ (also termed GHR-KO or Laron dwarf) male mice used in this study, developed by crossing 129Ola/BALB/c GHR^+/–^ animals (generously provided by Dr. J. J. Kopchick) with mice derived from crosses of C57BL/6 J and C3H/J strains, were produced in our breeding colony and maintained as a closed colony with inbreeding minimized by avoiding brother x sister matings. The animals were housed under temperature- and light-controlled conditions (20–23 °C, 12-hr light/12-hr dark cycle) until the age of 24 months, when the animals were sacrificed and the ovaries collected. GHR^–/–^ males were mated with heterozygous (GHR^+/–^) females to produce GHR^–/–^ mice [5].

#### Bovine GH transgenic (bGHTg) mice

Male phosphoenolpyruvate carboxykinase (PEPCK)-bGHTg male mice and their normal male siblings were originally produced by microinjecting the bGH structural gene fused with the promoter of the rat PEPCK gene into the pronuclei of fertilized mouse eggs [23]. The hemizygous Tg mice used in this study were produced by mating GH-Tg males with normal C57BL/6 x C3H F1 hybrid females. The animals were housed in temperature- and light-controlled conditions (20–23 °C, 12-hr light/12-hr dark cycle) until the age of 6 months, when the animals were sacrificed and the ovaries collected.

### Morphological analysis of ovarian tissue

Ovarian tissues were fixed in 10% buffered formalin and subsequently embedded in paraffin. The ovaries were sectioned at a thickness of 3 μm with a Microtome HM 325, and the sections were mounted on glass slides and counterstained with periodic acid, Schiff’s reagent (PAS), Mayer’s hematoxylin, and eosin. The slides were examined by light microscope (BX41 Olympus).

### Periodic acid Schiff (PAS) staining

The sections were deparafinized and rehydrated. The 0.5% periodic acid solution was applied for 10 min and after that the Schiff reagent for 15 min. Between each step the sections were rinsed in tap water for 5 min. In the end the section were counterstained in Mayer’s hematoxylin for 1 min, washed in tap water for 5 min, dehydrated and closed in mounting medium with coverslip.

### Hematoxylin and eosin (H&E) staining

For H&E staining the sections were deparaffinized and rehydrated. The hematoxylin was applied for 3 min and subsequently the sections were rinsed in tap water for 10 min. In the next step the eosin was applied for 30 sec. Finally, slides were washed in distilled water, dehydrated and closed in mounting medium with coverslip.

## Results

### Ovarian morphology in 2-year-old laron dwarf mice (with low plasma IGF-1 levels) and normal age-matched WT littermate controls

**Figure 1:**
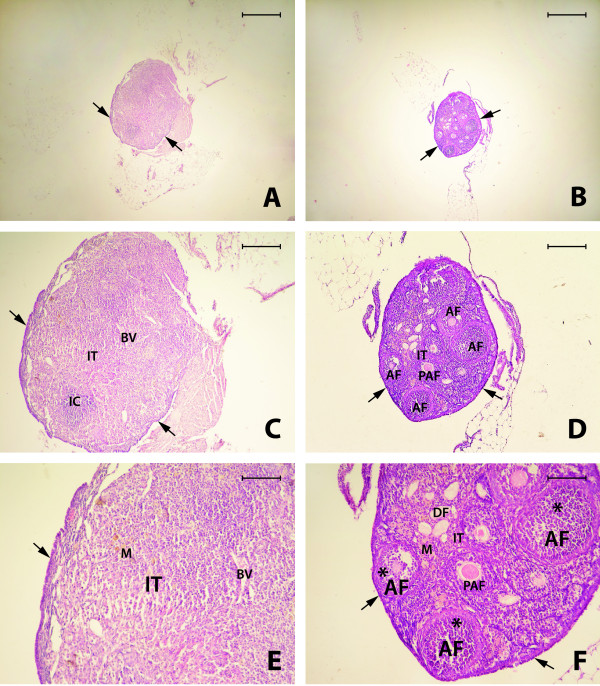
**Ovaries of 2-year-old wild type (WT) mice (A, C, E) and 2-year-old Laron dwarf (GHR/GHBP-KO) mice (B, D, F).** In WT mice, the ovarian follicles are missing. Cuboidal epithelium on the ovarian surface (arrow) and large numbers of interstitial cells (IT), blood vessels (BV), macrophages (M), and inflammatory cells (IC) are visible (A, C, E). In contrast, Laron dwarf mouse ovaries are very well developed and all types of follicles are present, including preantral (PAF), antral (AF), and degenerative (DF) follicles, and granulosa cells (asterix) are also visible. Well-organized cuboidal epithelium on the ovarian surface (arrow), macrophages (M), and interstitial tissue (IT) were also observed (B, D, F). H + E staining. Bar = 500μm (**A** and **B**), Bar = 200 μm (**C** and **D**), Bar = 100 μm (**E** and **F**)

The morphological structure of ovaries from normal 2-year-old WT mice exhibited a blurred border between cortex and medulla. The surface of the ovaries was covered by a simple cuboidal epithelium, and the ovarian cortex lacked the ovarian follicles observed in the ovaries of younger mice at reproductive age. Specifically, there were no visible primary, preantral, antral or Graffian follicles. The amount of interstitial tissue was increased compared with younger mice, and we observed inflammatory cells, macrophages, and blood vessels. (Figure 1A, C, and E). Furthermore, some ovarian sections from 2-year-old normal WT mice were found to contain large degenerative antral follicles that developed into cysts and small degenerative follicles in interstitial tissue (not shown). The cells in interstitial tissue were often surrounded by empty spaces that were remnants of degenerated granulosa cells and oocytes. In some of the ovaries, we observed numerous hypertrophied corpora lutea.

**Figure 2:**
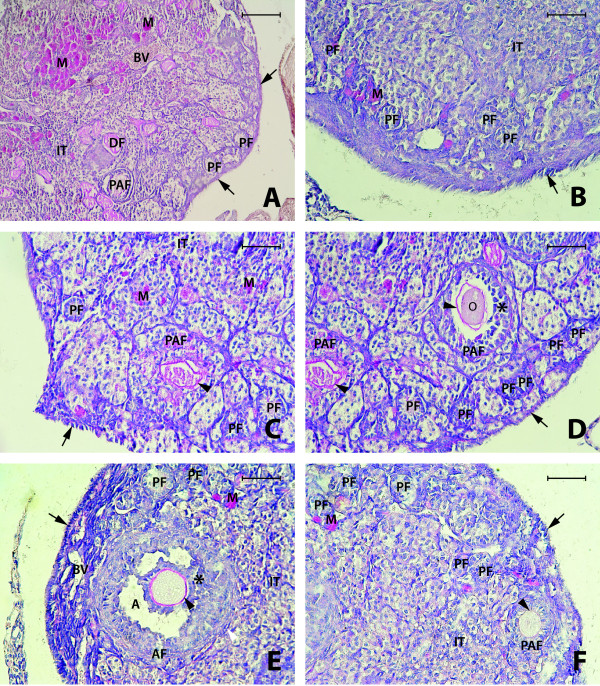
**Histochemical images of ovaries from 2-year-old Laron (GHR/GHBP-KO) mice (A, B, C, D, E, F).** Primary (PF), preantral (PAF), antral (AF), and degenerative (DF) follicles, as well as granulosa cells (asterix), theca cells (white arrowhead), and zona pellucida (black arrowheads) are visible. Cuboidal epithelium on the ovarian surface (arrow), as well as macrophages (M) and interstitial tissue (IT) are also visible. PAS staining. Bar = 100 μm (**A**), Bar = 50 μm (**B** – **F**)

By contrast, the ovaries of 2-year-old Laron dwarf mice were smaller in size and had a different morphology than ovaries from 2-year-old WT control mice. We found a regular cuboidal epithelium on the surface and, more importantly, morphological structures typical of ovaries seen in younger mice at reproductive age. Specifically, we observed primary, preantral, antral and Graffian follicles, and the interstitial cells were less numerous than in 2-year-old WT controls. At the same time, we observed some degenerative follicles and macrophages, and blood vessels were present in the medullary region of the ovary. Overall, the morphology of ovaries from 2-year-old Laron dwarf mice suggests that there is no ovarian failure (Figure 1B, D, and E and Figure 2).

### The morphology of ovaries in 0.5-year-old bGHTg mice (with high circulating plasma IGF-1 levels) and normal age-matched WT littermates

**Figure 3:**
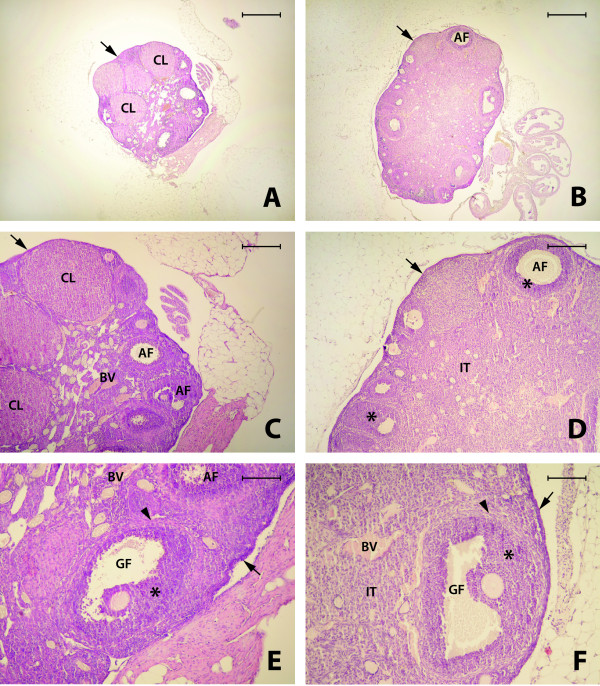
**Ovaries of 0.5-year-old wild type (WT) mouse (A, C, E) and 0.5-year-old bovine GH transgenic (bGHTg) mice (B, D, F).** All types of well-developed follicles, including preantral (PAF), antral (AF), Graffian (GF) follicles, as well as granulose cells (asterix) and theca cells (arrowhead) are visible in WT mouse ovaries. Numerous corpora lutea (CL), cuboidal epithelium on the surface (arrow), and blood vessels (BV) are also present (A, C, E). The ovaries from bovine GH transgenic mice (B, D, F) were bigger than ovaries from WT mice (A, C, E). All types of follicles were observed, including antral (AF) and Graffian (GF) follicles, and granulose cells (asterix) and theca cells (arrowhead) are also visible. In addition, corpora lutea (CL), cuboidal epithelium on the surface (arrow), interstitial tissue (IT), and blood vessels (BV) are visible (B, D, F). H + E staining. Bar = 500μm (**A** and **B**), Bar = 200 μm (**C** and **D**), Bar = 100 μm (**E** and **F**)

As expected, 0.5-year-old WT mice displayed normal ovarian morphology, including regular cuboidal epithelium on the surface, the presence of all types of follicles in the cortex (primary, preantral, antral, and Graffian), the presence of blood vessels in the medullary region of the ovaries, and a small amount of interstitial tissue. The corpora lutea were visible in some of the sections, and cells in these structures had brightly eosinophilic cytoplasm with centrally located nuclei (Figure 3A, C, and E).

In contrast, ovaries isolated from 0.5-year-old bGHTg mice were larger in size than ovaries isolated from age-matched WT littermates. The cuboidal epithelium on the surface was well developed and we observed the presence of follicles at different stages of development (primary, preantral, antral, and Graffian) that tended to be bigger than in normal control ovaries. The medullary regions of the ovaries were enriched in blood vessels, and we also observed an increase in interstitial tissue surrounding the follicles. Between the interstitial cells, we also found some degenerating follicles (Figure 3B, D, and F).

## Discussion

In our study we compared the morphology of the ovaries isolated from 2-year-old Laron mice (with low circulating plasma levels of IGF-1) and 0.5-year-old bGHTg mice (with high circulating plasma levels of IGF-1) with their normal age-matched littermates. The ages of Laron dwarf mutants employed in our studies were selected on the basis of their overall survival (_~_4 years for Laron dwarf mice and _~_1 year for bGHTg mice). Thus, Laron dwarf and bGHTg animals were investigated at approximately the midpoints of their respective lifespans.

Ovarian morphology of 2-year-old Laron dwarf mice exhibited significant differences compared with 2-year-old WT mice, which had all the signs of senescence. While ovaries in Laron dwarf mice were smaller, they showed a normal structure, including cuboidal epithelium on the surface and all types of ovarian follicles (primary, preantral, antral and Graffian follicles) in the cortex. However, we also observed some degenerative follicles and macrophages, which is unsurprising given the advanced age of these mice. The interstitial tissue was less pronounced compared with 2-year-old WT mice, and blood vessels and arteries were found in the central region of the medulla. Overall, the ovaries of Laron dwarf mice had a morphological structure similar to the structure of normal wild type mice at reproductive age [24,25], which suggests no significant ovarian failure in these mice. In support of this conclusion, we observed that some 2-year-old Laron dwarf mice can became pregnant and deliver life off spring (unpublished data). The lower biological age of ovaries in Laron dwarf mice and their continued fertility are, likely due to delayed aging [26].

While we investigated ovaries from 2-year-old Laron dwarf mice, the ovaries from these animals have also been studied extensively at younger ages. For example, Slot et al. [6] reported that ovaries from 9-week-old Laron dwarf mice contain more primordial follicles than WT mice. Bachelot et al. [4] studied reproductive system morphology in 10-week-old Laron dwarf mice and found that, despite normal structure, the number of follicles in ovaries from these animals was reduced. Furthermore, the ovulation rate was reduced and was not increased after gonadotropin stimulation, which indicates an ovarian defect rather than deficiency in gonadotropins. The intraovarian expression of IGF-I mRNA was similar to that found in WT mice. Interestingly, 18-month-old mice were able to reproduce, which suggests prolonged ovarian function [4]. In other reports [5,11], Laron dwarf females were found to be fertile, but the estrous cycle was irregular. The number of pre-ovulatory follicles and corpora lutea and the ovulation and implantation rates were also reported to be reduced [5,11].

The origin of oocytes in ovaries at advanced ages is still somehow controversial. It has been demonstrated that ovaries even at advanced age contain a population of primitive embryonic-like stem cells that can potentially give rise to oocytes [27-32]. These cells have been assigned different names in the literature and could be related as postulated by Bhartiya [28] and Virant-Klun [29] to a population of so-called very small embryonic-like stem cells (VSELs). In support of this hypothesis, our recent studies demonstrated that Laron dwarf mice have increased numbers of these cells in BM [33,34]. Thus, it is important to see whether the number of VSELs is increased in the ovaries of Laron dwarf mice.

The changes that we observed in ovaries of 2-year-old WT littermates, such as a blurred border line between cortex and medulla, large degenerative antral follicles developing into cysts, small degenerative follicles admixed with interstitial tissue, an increase in interstitial tissue, hypertrophic corpora lutea, numerous inflammatory cells and macrophages, and, most important, a lack of ovarian follicles that are observed at reproductive age, were similar to those described in the literature [25,35]. Laszczyńska et al. [36] observed similar changes in the ovaries of postmenopausal women.

In contrast to Laron dwarf mice, in bGHTG mice (with high circulating plasma IGF-1 levels) the ovarian and follicle dimensions were larger. This could be explained as a response to bGH, which leads to hypertrophy of several organs, including bones, skeletal muscle, heart, liver, and spleen [19,20,37]. Danilovich et al. [15] reported that high level of GH protects granulosa cells from apoptosis and reduces follicular atresia. Cecim et al. [38,39] found that bGHTg mice show accelerated prepubertal somatic growth and sexual maturation, but that the mating and pregnancy rates are reduced. Similar changes in fertility were observed in normal mice exposed to a prolonged series of bGH injections [38,39]. The suppression of female fertility in bGHTg mice is proportional to plasma GH level [21,22], and the elevated bGH in plasma could therefore explain the reduced fertility due to luteal failure and reduced progesterone levels during early pregnancy. Since the injection of progesterone enhanced the rate of pregnancy in bGHTg mice, the observed luteal failure is probably caused by inadequate prolactin (PRL) secretion, as injections of PRL significantly increased pregnancy rates in transgenic female mice [38,39].

One has also take into consideration that despite high level of IGF-1 in bGHTg mice, IGF-1 may not be unavailable to the ovaries due to the high circualting IGFBP1 level that has a high binding affinity for IGF-1 and could sequester it – thus reducing bio-availability of IGF-1 to various tissues [40]. This could explain the normal appearing ovarian morphology despite high levels of IGF-1 in bGHGTg mice at age of 6 month.

Overall, our observations indicate that decreased fertility in transgenic bGH mice is not caused by morphological abnormalities. We also did not observe malignant transformation of ovarian tissue; however, this could be explained by the relatively small group of mice involved in this study [41]. We expect to see more changes in bGHT mice at the age of approximately 1 year.

## Conclusions

Morphological analysis revealed a lower biological age of ovaries isolated from 2-year-old Laron dwarf mice compared with ovaries from normal WT littermates at the same age. At the same time, no morphological features of accelerated aging were found in 0.5-year-old bGHTg mice.

## Competing interests

The all authors declare that they have no competing interests.

## Authors’ contribution

SS-G: performed morphological and histochemical analysis of ovarian sections, found the result and wrote the manuscript. ML: helped in planning and supervised the work, participated in morphological and histochemical analysis of ovarian sections and helped in writing the manuscript. KP: participated in results analysis and helped in writing the manuscript. WG: helped in morphological and histochemical analysis of ovarian sections, found the result and correct the manuscript. JJK: Provided Laron dwarf mice, approved manuscript. AB: Provided bGH-Tg mice and approved a final version of manuscript. MK: obtained the ovarian tissue. MZR: helped in planning and supervised the work, helped in writing the manuscript and corrected the final version of manuscript. All authors have read and approved the final manuscript.
